# Contagious and Infectious Diseases

**Published:** 1884-07

**Authors:** 


### Book Reviews.

---

CONTAGIOUS AND INFECTIOUS DISEASES; Measures for their Prevention and arrest—Small-Pox (Variola;) Modified Small-Pox (Varioloid); Chicken-Pox (Varicella); Cow-Pox (Variolæ Vaccinæ). Vaccination, Spurious Vaccination, illustrated by eight colored plates.

Circular No. 2 prepared for the guidance of the Quarantine Officers and Sanitary Inspectors of the Board of Health of the State of Louisiana. By Joseph Jones, M. D., President of the Board of Health of the State of Louisiana.

The above is an archeological museum of eruptive diseases which to be appreciated requires an intelligent observer in like manner as in an examination of the rare specimens from Pompeii, at Naples, the Egyptian mummies at Cairo, or the collections in the tower of London.

Whether the officers for whom these important data are gathered together shall give them the serious consideration they deserve remains to be seen, but in any event the medical profession throughout the United States must realize their obligations to the indefatigable zeal of Dr. Jones, in recovering from the records of the past history of vaccination, such valuable materials as are contained in this volume.

There are, perhaps, few medical men who have a thorough acquaintance with the details of the incipiency and development of this discovery by Dr. Jenner, which has revolutionized the progress of corrective measures for small-pox; and it is a very prevalent error that the vaccine virus is the modification impressed upon the variolous poison by being communicated to the cow, and thus mitigated in its virulence so as to be innocuous when introduced into the human system.

Those who have a desire, as all should havé, to learn the facts connected with the origin of the vaccine matter, should read carefully the painstaking investigations, with the report of cases, presented in the almost forgotten publications of Jenner, Pearson, Woodville, Ceely, and others, not omitting the triumphant refutations by Ring, of the antagonistic allegations of Rawley, Mosely, Squirul & Co., who attempted to prejudice the minds of the people against vaccination by the most gross misrepresentations.

The battle that was waged at that time is being fought over again at the present day, not by men of science in the opposition, but by the ill-advised and ignorant class both in England and America, so that imprisonment and fines are necessary in Great Britain to enforce the compulsory vaccination laws, and all sorts of temporary measures are resorted to in this country to prevail upon those directly interested to accept the free gift of immunity from small-pox.

A great part of the indisposition to vaccination results doubtless, from the occasional disastrous effects of spurious vaccination, which from want of proper precautions, has entailed diseases upon the recipients, far worse than even the genuine small-pox. But the abuses growing out of any sanitary proceeding cannot be justly held as an objection to its proper observances, and all who have discretion should instead of rejecting this salutary measure, set about eliminating the injurious contamination and secure the full benefit of the pure vaccine virus.

It is evident not only from the experience of military surgeons amongst ourselves, but from the array of observations made by prominent medical men in different parts of the world, that disease is capable of being transmitted jointly with the vaccine lymph from one individual to another, and that the special derangements of the system in the person receiving the virus, may complicate and aggravate its influence in a very characteristic manner. But it is on the other hand clearly established that while variola intensifies or even develops predisposition to certain diseases, vaccine mitigates and corrects the tendency to those diseases. It thus has a decided vantage ground over the old plan of inoculation, in not only being milder in its operation, but in modifying the general organism as an alterant so as to eliminate constitutional proclivities to scrofulous and cutaneous affections, while, at the same time, it proves even more effectual against the variolous susceptibility than an attack of small-pox. There are many well established instances of the recurrence of genuine small pox after the subject had been repeatedly exposed to contagion, and the writer has verified this repetition of this disease with a fatal result.

The peculiarities or idiosyncrasies of these individuals should not, however, militate against the general laws which control the organization under diseased action.

The transition state of our knowledge as to the phenomena that complicate inflammatory conditions of the different tissues, and the corresponding measures of treatment, renders it necessary to observe closely the facts presented in practice. The clinical data collected with patient attention to all the details of cases, whether of the so-called idiopathic nature or the result of accidents, must elucidate the vexed problem of the source of disease in the organism. While physiological and pathological experiments aid in reaching correct conclusions, it strikes me forcibly that there is too great a tendency at the present day to ignore the facts of clinical observations; and the records of experience are set aside by hypothesis and preconceived theory as to the inflammatory process set up in the tissues. It is vastly more important to determine the precise nature of the changes or abnormalties than to fix definitely the form of the entity which originates the trouble, since similar results may proceed from various sources. Whether the disturbance of the natural relations of the elements that makes up the healthy organization comes from without or within, does not influence so much the means of cure as the accurate knowledge of the effects produced upon the parts involved, or in other words a thorough acquaintance with the proximate cause of disease. The antecedents or even concomitants are not always material factors of abnormalty.

The remedy for preventing small-pox being recognized in the influence of vaccine when pure, we should not discard it because it may from accidental causes become mixed with impurities, but our efforts should rather be directed to obtaining the unadulterated virus, and means should be adopted to secure all interested against any miscarriage in this respect.

If those charged with sanitary regulations are provided with the means of propagating the cow-pox from original sources amongst healthy children, it devolves upon them to supply such guranteed matter to all who vaccinate, and no trouble or expense should be spared in thus ridding the country of spurious vaccination.

The immense sums of money that have been expended and continue to be expended by those in authority for treating small-pox patients at the public expense, might more appropriately be paid out for preventive means, and let pure vaccine be secured at any cost as the initiatory step.

As all mankind are influenced to a greater or less extent by self interest, it might prove as a stimulus to medical men even of the noblest and purest sentiments, who are assigned to the post of vaccinating, to be paid per capitum for all the cases that are successfully vaccinated, and make it obligatory upon them in this way to observe and report the result. Such an inducement would doubtless prompt them to bring all their arguments in favor of vaccination to bear upon the parents of children and insure a careful performance of this simple yet important operation.

